# Supplementary material for: A Combined Near-Infrared and Mid-Infrared Spectroscopic Approach for the Detection and Quantification of Glycine in Human Serum
Source: Sensors (Basel). 2022 Jun 15;22(12):4528. doi: 10.3390/s22124528 (PMC9228712; doi:10.3390/s22124528)
Supplement: Supplementary file 1 [file sensors-22-04528-s001.zip › sensors-1736409-supplementary.pdf]

Supplementary Information

Near-Infrared Combined with Mid-Infrared Spectroscopic Detection of Low-Molecular Weight Proteins in Human Serum Using Glycine as a Model Protein

Thulya Chakkumpulakkal Puthan Veettil [1,2] and Bayden R. Wood [1,*]

[1] Centre for Biospectroscopy, Monash University, Clayton, VIC 3800, Australia; thulya.chakkumpulakkalputhanveettil@monash.edu

[2] Centre for Sustainable and Circular Technologies (CSCT), University of Bath, Bath BA2 7AY, UK

* Correspondence: bayden.wood@monash.edu

Spectral Analysis of spiked serum samples: ATR spectroscopy

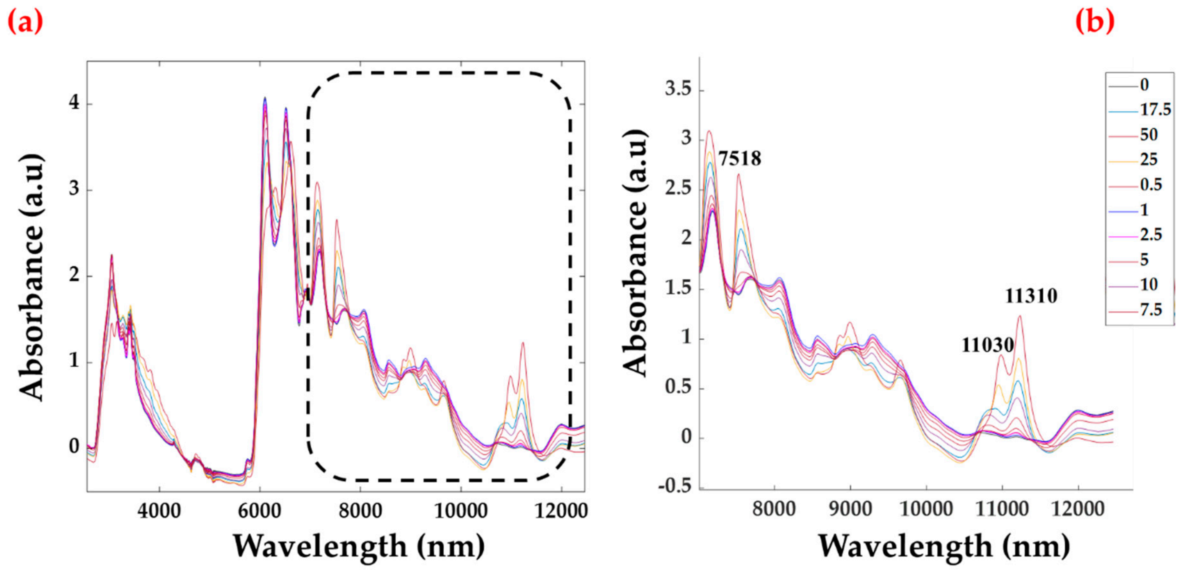

Figure S1. a) Baseline corrected raw spectra of glycine spiked human plasma samples. The concentration of glycine is ranging from 0 – 50 mg/ml. **b)** magnified view of 12000 – 7000 nm region where major contribution from glycine is observed.

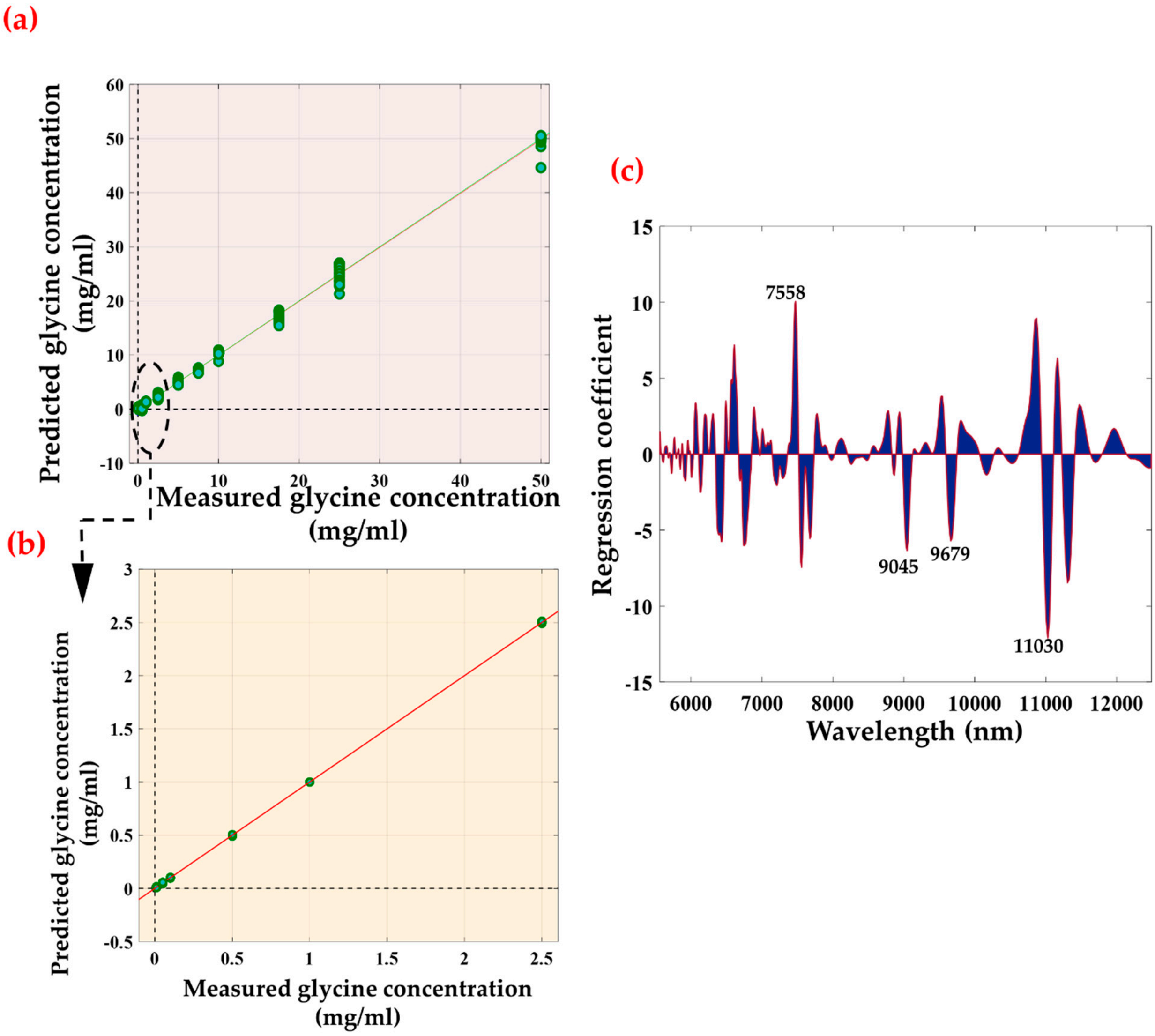

Figure S2. PLS – regression plot and its associated regression vector in the 12500 – 5555 nm region.

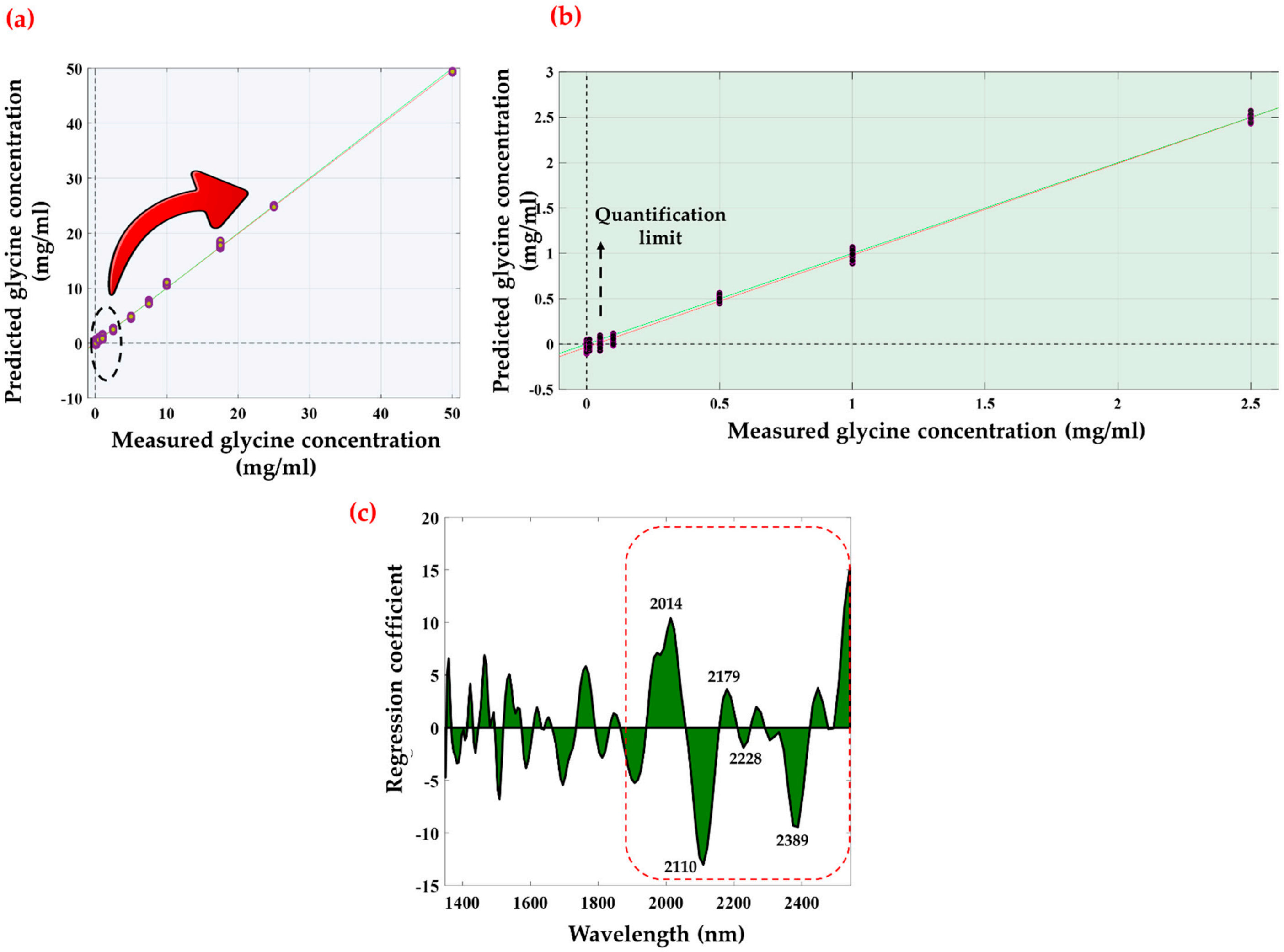

Figure S3. PLS – regression plot and its associated regression vector in the 2500 – 1350 nm region.

Table S1. Major band positions observed from the NIR and MIR spectra.

**Band**	**Band Assignment**
906 cm^{-1}	CH_2 rocking from glycine
1036 cm^{-1}	C– N stretching from glycine
1111 cm^{-1}	$N–H_3$ rocking from glycine
1330 cm^{-1}	C H_2 wagging from glycine
1414 cm^{-1}	O–C=O symmetric stretching from glycine from glycine
2055 nm	N—H stretch/amide Ib combination band
2110 nm	(N-H + C-H) combination band from glycine
2180 nm	N—H bend second overtone, C—H stretch/C==O stretch combination

	and C==O stretch/amide IIIb combination mode
2204 nm	combinations of (C-H and C-H) stretching modes from glycine
2375 nm	combinations of (C-H and C-H) stretching modes from glycine

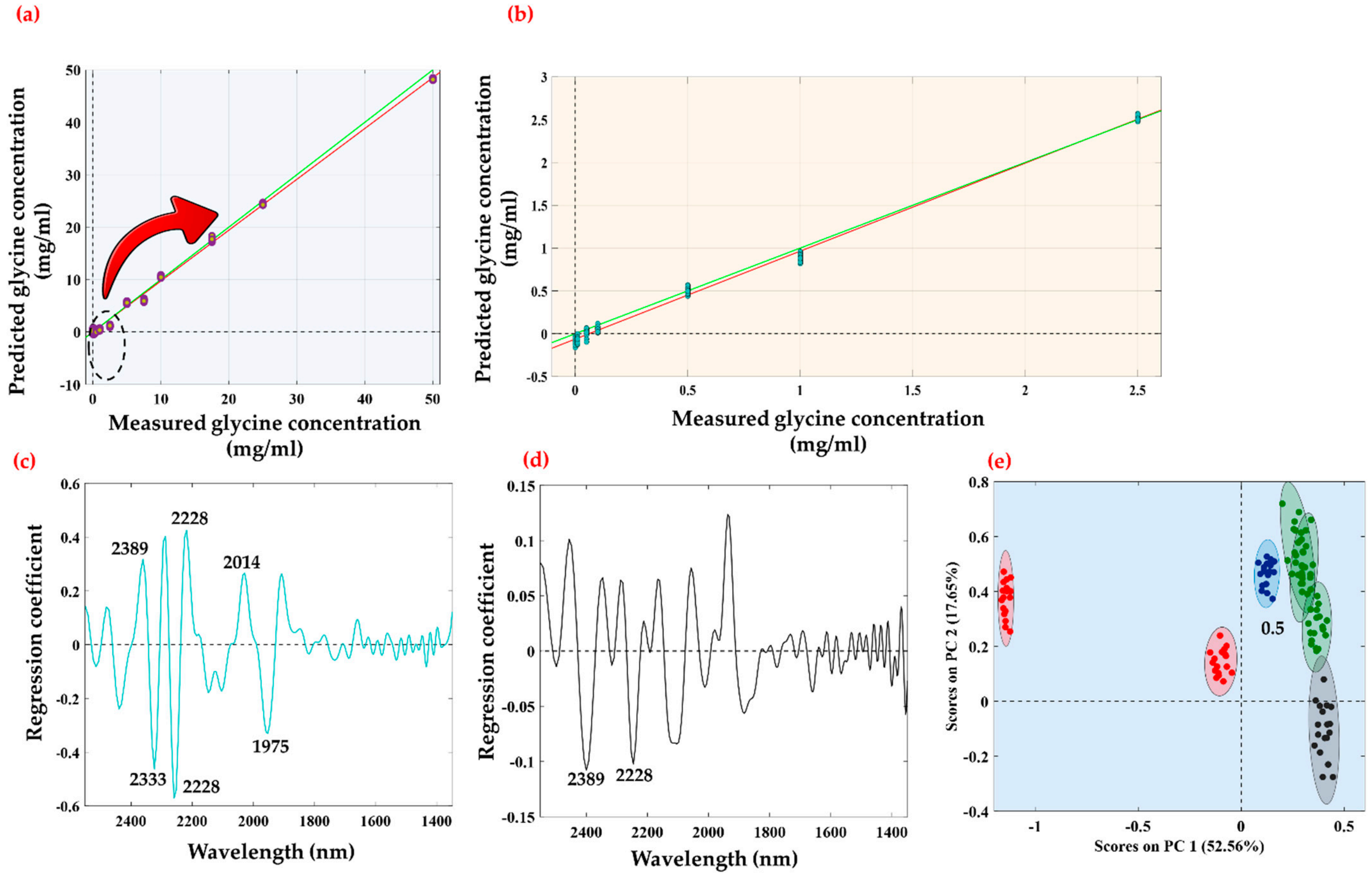

Figure S4. PCR predicted model of glycine spiked serum samples and it's a) regression plot of entire range between 0 (control) – 50 mg/mL region b) remodelled 0 – 2.5 mg/mL c) corresponding regression coefficient/vector for 0 – 50 mg/mL d) 0 – 2.5 mg/mL over 2500 – 2000 nm region. e) corresponding PC scores plot.

Table S2. Values associated with the LoB and LoD calculation from the regression statistics.

Method	$Mean_{blank}$	SD_{blank}	LoB	$SD_{low\ concentration}$	LoD
ATR	-0.187	0.132	0.030	0.142	0.265
NIR	-0.032	0.129	0.181	0.0288	0.228
Combined ATR - NIR	-0.217	0.1536	0.0354	0.0821	0.170
